# Supplementary material for: Validation of a polygenic risk score for frailty in the Lothian Birth Cohort 1936 and English longitudinal study of ageing
Source: Sci Rep. 2024 Jun 1;14:12586. doi: 10.1038/s41598-024-63229-y (PMC11143351; doi:10.1038/s41598-024-63229-y)
Supplement: Supplementary file 1 — Supplementary Information 1. [file 41598_2024_63229_MOESM1_ESM.docx]

Validation of a polygenic risk score for Frailty in the Lothian Birth Cohort 1936 and English Longitudinal Study of Ageing

**Data preparation**

This project was a university project, similar to an MSc dissertation which was cleared by the Advanced Care Research centre and Lothian Birth Cohorts team, the analysis was then developed into this study – a preregistration was not published. In addition to the quality control measures performed on the base and target datasets, the phenotype data (outcome data) had to be cleaned and categorised. Missing data were screened to ensure that any missing values were coded as NA before being inputted into PRSice. To create the polygenic scores in LBC1936, data were then sorted into ten different datasets. Five sets included only the frailty scores of individuals from LBC1936 at each wave – this was then coded into PRSice under the pheno – command. The other five datasets were age, sex and principal components at each wave. Sex and principal components remained consistent, only age differed. The latter five datasets were coded into PRSice under the cov- function.

**PRSice sample script from Wave 1 of LBC1936**

The script created to create the polygenic risk scores and run the multiple linear regressions model – this is an example for the PRS and the regressions ran for LBC1936 wave 1.

--A1 Effect_allele \

--A2 Other_allele \

--bar-levels 0.05,0.01,0.1,0.5,1 \

--base Basefrailty.txt \

--beta \

--binary-target F \

--bp Position \

--chr Chromosome \

--pvalue P_value \

--clump-kb 250kb \

--clump-p 1.000000 \

--clump-r2 0.100000 \

--cov covmerged.txt \

--cov-col Sex,Age,@C[1-4] \

--cov-factor Sex \

--fastscore \

--ignore-fid \

--num-auto 22 \

--out prs.score \

--score avg \

--pheno phenof1.txt \

--seed 270497445 \

--print-snp \

--snp Markername\

--stat BETA \

--target LBC36.target

**Sampling the ELSA data**

ELSA participants, across all five groups, came from 9 collection points (known as waves in ELSA) from Wave 1 2002/2003– to Wave 9 2018/2019. The tables below shows the split of participants across the waves and the mean frailty scores and standard deviations at each wave.

Table S1 showing the spread of group 1 across ELSA wave collection points.


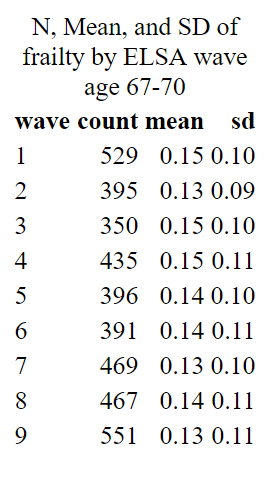


Table S2 showing the spread of group 2 across ELSA wave collection points.

**
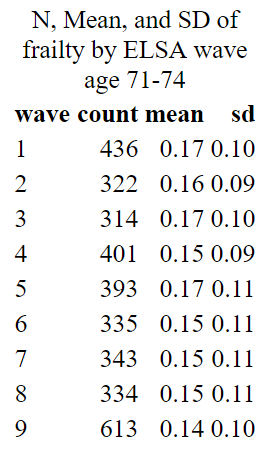
**

Table S3 showing the spread of group 3 across ELSA wave collection points.


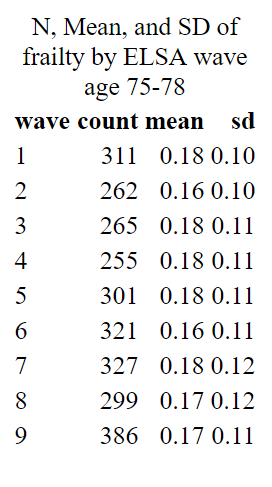


Table S4 showing the spread of group 4 across ELSA wave collection points.


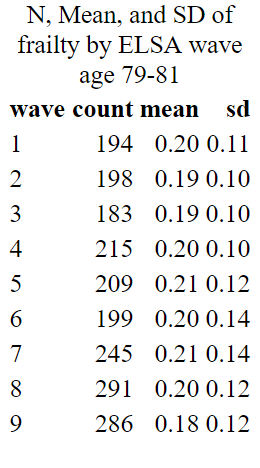


Table S5 showing the spread of group 5 across ELSA wave collection points.


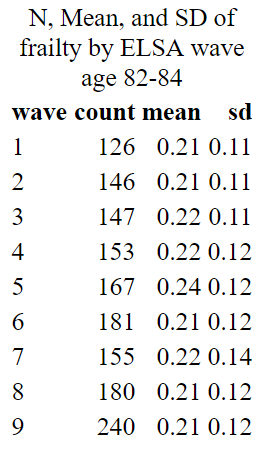


Table S6. 1. Numbers of participants who had a previous frailty index measure across the age groups vs participants who only had a single measure at each age group in ELSA.

| Group | Longitudinal participants | Participants with no previous frailty measure | Total |
| --- | --- | --- | --- |
| 1/Baseline | 3983 | NA | 3983 |
| 2 | 2812 | 679 | 3491 |
| 3 | 2322 | 405 | 2727 |
| 4 | 1791 | 229 | 2020 |
| 5 | 1345 | 150 | 1495 |
|  |  |  |  |

**Frailty indexes across cohorts**

**Supplementary Table S7: Elements comprising the frailty index in UK Biobank** taken from reference 15 - Atkins JL, Jylhävä J, Pedersen NL, Magnusson PK, Lu Y, Wang Y, Hägg S, Melzer D, Williams DM, Pilling LC. A genome‐wide association study of the frailty index highlights brain pathways in ageing. Aging Cell. 2021 Sep;20(9):e13459.

|  |  |  |  |  |  |
| --- | --- | --- | --- | --- | --- |
| **Type of deficit** | **Item** | **Trait** | **Categories** | **Coding in FI item** | **Score of 1 n (%)†** |
| *Sensory* | 1 | Glaucoma * | no,yes | Categorised 0/1 | 3,728 (2.26) |
|  | 2 | Cataracts * | no,yes | Categorised 0/1 | 8,993 (5.46) |
|  | 3 | Hearing difficulty | no, yes, completely deaf | Categorised 0/1 (combined yes/deaf groups as 1) | 52,506 (31.90) |
| *Cranial* | 4 | Migraine * | no,yes | Categorised 0/1 | 3,863 (2.35) |
|  | 5 | Dental problems | ulcers, painful gums, bleeding gums, loose teeth, toothache, dentures | Categorised 0/1 for none vs. any | 71,494 (43.43) |
| *Mental wellbeing* | 6 | Self-rated health | excellent, good, fair, poor | 0 – excellent; 0.25 – good; 0.5 - fair; 1 – poor | 6,231 (3.79) |
|  | 7 | Fatigue: frequency of tiredness / lethargy in last two weeks | not at all, several days, more than half, nearly every day | 0, 0.25, 0.5, 1, respectively | 6,928 (4.21) |
|  | 8 | Sleep: experience of sleeplessness/insomnia | never/rarely, sometimes, usually | Categorised 0, 0.5, 1, respectively | 49,304 (29.95) |
|  | 9 | Depressed feelings: frequency in last two weeks | not at all, several days, more than half, nearly every day | 0 – not at all, 0.5 – several days, 0.75 - more than half, 1 – nearly every day | 1,834 (1.11) |
|  | 10 | Self-described nervous personality | no, yes | Categorised 0/1 | 35,478 (21.55) |
|  | 11 | Severe anxiety/ panic attacks * | no, yes | Categorised 0/1 | 1,936 (1.18) |
|  | 12 | Common to feel loneliness | no, yes | Categorised 0/1 | 24,005 (14.58) |
|  | 13 | Sense of misery (ever/never) | no, yes | Categorised 0/1 | 57,738 (35.08) |
| *Infirmity* | 14 | Infirmity: long-standing illness or disability | no, yes | Categorised 0/1 | 60,831 (36.95) |
|  | 15 | Falls in last year | categorical: no falls, one fall, more than one | 0, 0.5, 1, respectively | 10,831 (6.58) |
|  | 16 | Fractures/broken bones in last five years | no, yes | Categorised 0/1 | 15,881 (9.65) |
| *Cardiometabolic* | 17 | Diabetes * | no, yes | Categorised 0/1 | 10,881 (6.61) |
|  | 18 | Myocardial infarction * | no, yes | Categorised 0/1 | 6,221 (3.78) |
|  | 19 | Angina * | no, yes | Categorised 0/1 | 8,779 (5.33) |
|  | 20 | Stroke * | no, yes | Categorised 0/1 | 3,756 (2.28) |
|  | 21 | High blood pressure * | no, yes | Categorised 0/1 | 59,258 (36.00) |
|  | 22 | Hypothyroidism * | no, yes | Categorised 0/1 | 9,586 (5.82) |
|  | 23 | Deep-vein thrombosis * | no, yes | Categorised 0/1 | 4,450 (2.70) |
|  | 24 | High cholesterol * | no, yes | Categorised 0/1 | 45,052 (27.37) |
| *Respiratory* | 25 | Breathing: wheeze in last year | no, yes | Categorised 0/1 | 34,566 (21.00) |
|  | 26 | Pneumonia * | no, yes | Categorised 0/1 | 2,800 (1.70) |
|  | 27 | Chronic bronchitis/emphysema * | no, yes | Categorised 0/1 | 3,839 (2.33) |
|  | 28 | Asthma * | no, yes | Categorised 0/1 | 17,447 (10.60) |
| *Musculoskeletal* | 29 | Rheumatoid arthritis * | no, yes | Categorised 0/1 | 2,314 (1.41) |
|  | 30 | Osteoarthritis * | no, yes | Categorised 0/1 | 19,804 (12.03) |
|  | 31 | Gout * | no, yes | Categorised 0/1 | 3,208 (1.95) |
|  | 32 | Osteoporosis * | no, yes | Categorised 0/1 | 4,187 (2.54) |
| *Immunological* | 33 | Hayfever, allergic rhinitis or eczema * | no, yes | Categorised 0/1 | 32,727 (19.88) |
|  | 34 | Psoriasis * | no, yes | Categorised 0/1 | 1,763 (1.07) |
| *Cancer* | 35 | Any cancer diagnosis * | no, yes | Categorised 0/1 | 19,068 (11.58) |
|  | 36 | Multiple cancers diagnosed (number reported) | Range from 0 to 6 | 0 - no cancer or single cancer, 1 - multiple cancers | 1,430 (0.87) |
| *Pain* | 37 | Chest pain | no, yes | Categorised 0/1 | 25,041 (15.21) |
|  | 38 | Head and/or neck pain | no, yes (combining responses to pain in head and neck/shoulders) | Categorised 0/1 | 49,029 (29.78) |
|  | 39 | Back pain | no, yes | Categorised 0/1 | 40,764 (24.76) |
|  | 40 | Stomach/abdominal pain | no, yes | Categorised 0/1 | 10,703 (6.50) |
|  | 41 | Hip pain | no, yes | Categorised 0/1 | 21,192 (12.87) |
|  | 42 | Knee pain | no, yes | Categorised 0/1 | 37,661 (22.88) |
|  | 43 | Whole-body pain | no, yes | Categorised 0/1 | 2,468 (1.50) |
|  | 44 | Facial pain | no, yes | Categorised 0/1 | 2,402 (1.46) |
|  | 45 | Sciatica * | no, yes | Categorised 0/1 | 1,643 (1.00) |
| *Gastrointestinal* | 46 | Gastric reflux * | no, yes | Categorised 0/1 | 8,371 (5.09) |
|  | 47 | Hiatus hernia * | no, yes | Categorised 0/1 | 5,076 (3.08) |
|  | 48 | Gall stones * | no, yes | Categorised 0/1 | 3,593 (2.18) |
|  | 49 | Diverticulitis * | no, yes | Categorised 0/1 | 2,748 (1.67) |
|  |  |  |  |  |  |
|  |  | FI score as previously validated in UK Biobank by Williams et al^28^. | |  |  |
|  |  | †N=164,610 (60-70 year olds; European descent; complete case analysis of all 49 FI components). | |  |  |
|  |  | * Participants reported medically diagnosed conditions for these items. | |  |  |
|  |  |  |  |  |  |

| **Supplementary Table ST8: Elements comprising the frailty index in LBC1936** created  by 2nd author M Welstead. |
| --- |

| **Items** | **Coding** | **Cut offs based on** |
| --- | --- | --- |
| Systolic Blood Pressure | <5^th^ percentile (1), 5^th^-20^th^ percentile (0.5), >20^th^ percentile (0) | Recommended technique (Theou et al., 2015) |
| Diabetes | Yes (1) or No (0) | Already binary variable |
| High Cholesterol | Yes (1) or No (0) | Already binary variable |
| Heart problem | Yes (1) or No (0) | Already binary variable |
| Stroke or mini stroke | Yes (1) or No (0) | Already binary variable |
| Crampy pains in calves | Yes (1) or No (0) | Already binary variable |
| Problems with blood circulation | Yes (1) or No (0) | Already binary variable |
| Thyroid Disorder | Yes (1) or No (0) | Already binary variable |
| Cancer | Yes (1) or No (0) | Already binary variable |
| Parkinson’s disease | Yes (1) or No (0) | Already binary variable |
| Dementia | Yes (1) or No (0) | Already binary variable |
| Arthritis | Yes (1) or No (0) | Already binary variable |
| Any other chronic disease | Yes (1) or No (0) | Already binary variable |
| Polypharmacy | >4 medications (1), ≤4 medications (0) | Recommended technique (Theou et al., 2013) |
| Body Mass Index | 18.5 to <25 (0), 25 to <30 (0.5), <18.5 or >equal to 30 (1) | Recommended technique (Chamberlain, Sauver, et al., 2016) |
| 6m walk time (gait speed) | >10 seconds or physically unable (1), <10 seconds (0) | Recommended technique (Hoogendijk et al., 2017) |
| Able to stand up from a chair | Yes (1) or No (0) | Already binary variable |
| Grip strength (strongest hand and stratified by sex and BMI) | <5^th^ percentile (1), 5^th^-20^th^ percentile (0.5), >20^th^ percentile (0) | Recommended technique (Theou et al., 2015) |
| Townsend Disability Scale | 11 – 18 (1), 0 -10 (0) | Recommended technique (Fiona Elaine Matthews et al., 2016) |
| Peak Expiratory Flow rate (stratified by sex) | <5^th^ percentile (1), 5^th^-20^th^ percentile (0.5), >20^th^ percentile (0) | Recommended technique (Theou et al., 2015) |
| Forced expiratory volume (stratified by sex) | <5^th^ percentile (1), 5^th^-20^th^ percentile (0.5), >20^th^ percentile (0) | Recommended technique (Theou et al., 2015) |
| Depression | 11 -21 (1), 8 – 10 (0.5), 0 – 7 (0) | Recommended technique (Zigmond & Snaith, 1983) |
| Anxiety | 11 -21 (1), 8 – 10 (0.5), 0 – 7 (0) | Recommended technique (Zigmond & Snaith, 1983) |
| MMSE | <10 (1), 11-17 (0.75), 18 – 20 (0.5), 20 – 24 (0.25), >24 (0) | Recommended technique (Searle et al., 2008) |
| Digit Symbol | <5^th^ percentile (1), 5^th^-20^th^ percentile (0.5), >20^th^ percentile (0) | Recommended technique (Theou et al., 2015) |
| Block Design | <5^th^ percentile (1), 5^th^-20^th^ percentile (0.5), >20^th^ percentile (0) | Recommended technique (Theou et al., 2015) |
| Verbal Fluency | <5^th^ percentile (1), 5^th^-20^th^ percentile (0.5), >20^th^ percentile (0) | Recommended technique (Theou et al., 2015) |
| Matrix Reasoning | <5^th^ percentile (1), 5^th^-20^th^ percentile (0.5), >20^th^ percentile (0) | Recommended technique (Theou et al., 2015) |
| Reaction time test | <5^th^ percentile (1), 5^th^-20^th^ percentile (0.5), >20^th^ percentile (0) | Recommended technique (Theou et al., 2015) |
| Delayed recall | <5^th^ percentile (1), 5^th^-20^th^ percentile (0.5), >20^th^ percentile (0) | Recommended technique (Theou et al., 2015) |

| **Supplementary Table ST9**  **: Elements comprising the frailty index score and their seven domains in ELSA** taken from reference 16 - 16. Mekli, K. et al. Frailty Index associates with GRIN2B in two representative samples from the United States and the United Kingdom. PLoS ONE 13, e0207824; 10.1371/journal.pone.0207824 (2018). | | | | |  |  |  |
| --- | --- | --- | --- | --- | --- | --- | --- |
| **Variable name** | **Domain (taken from elsa questionnaire)** | **Description** |  |  |  |  |  |
| hemobwa | mobility | Difficulty walking 100m |  |  |  |  |  |
| hemobsi | mobility | Difficulty sitting 2 hrs |  |  |  |  |  |
| hemobch | mobility | difficulty getting up from chair after sitting long periods | | | |  |  |
| hemobcs | mobility | Difficulty climbing several flights of stairs without resting | | | |  |  |
| hemobcl | mobility | Difficulty climbing one flight of stairs without resting | | | |  |  |
| hemobst | mobility | Difficulty stooping, kneeling or crouching | | |  |  |  |
| hemobre | mobility | Difficulty reaching or extending arms above shoulder level | | | |  |  |
| hemobpu | mobility | Difficulty pulling or pushing large objects | | |  |  |  |
| hemobli | mobility | Difficulty lifting or carrying weights over 10 pounds (4.54kg) | | | |  |  |
| hemobpi | mobility | Difficulty picking up a 5p coin from a table | | |  |  |  |
| headldr | activities of daily life | Difficulty dressing, including putting on shoes and socks | | | |  |  |
| headlwa | activities of daily life | difficulty walking across a room | |  |  |  |  |
| headlba | activities of daily life | Difficulty bathing or showering | |  |  |  |  |
| headlea | activities of daily life | Difficulty eating, such as cutting up food | | |  |  |  |
| headlbe | activities of daily life | Difficulty getting in and out of bed | |  |  |  |  |
| headlwc | activities of daily life | Difficulty using the toilet including getting up or down | | | |  |  |
| headlma | activities of daily life | Difficulty using map to figure out how to get around strange place | | | | |  |
| headlpr | activities of daily life | Difficulty preparing a hot meal | |  |  |  |  |
| headlsh | activities of daily life | Difficulty shopping for groceries | |  |  |  |  |
| headlph | activities of daily life | Difficulty making telephone calls | |  |  |  |  |
| headlme | activities of daily life | Difficulty taking medications | |  |  |  |  |
| headlhg | activities of daily life | Difficulty doing work around the house or garden | |  |  |  |  |
| headlmo | activities of daily life | Difficulty managing money, eg paying bills, keeping track of  expenses | | | | |  |
| hedimbp | CV | High bp dx |  |  |  |  |  |
| hediman | CV | Angina dx |  |  |  |  |  |
| hedimmi | CV | Heart attack |  |  |  |  |  |
| hedimhf | CV | Congestive heart failure |  |  |  |  |  |
| hedimar | CV | Abnormal heart rhythm |  |  |  |  |  |
| hedimdi | CV | Diabetes or high blood sugar | |  |  |  |  |
| hedimst | CV | Stroke dx |  |  |  |  |  |
| hediblu | Chronic | Lung disease dx |  |  |  |  |  |
| hedibas | Chronic | Asthma dx |  |  |  |  |  |
| hedibar | Chronic | Arthritis dx |  |  |  |  |  |
| hedibos | Chronic | Osteoporosis |  |  |  |  |  |
| hedibca | Chronic | Cancer dx |  |  |  |  |  |
| hedibpd | Chronic | Parkinson's dx |  |  |  |  |  |
| hedibps | Chronic | Psychiatric condition |  |  |  |  |  |
| hedibad | Chronic | Alzheimer's dx |  |  |  |  |  |
| hedibde | Chronic | Dementia dx |  |  |  |  |  |
| psceda | Psych | Whether felt depressed much of the time during the past week | | | | |  |
| pscedb | Psych | Whether felt everything they did during the past week was an effort | | | | |  |
| pscedc | Psych | Whether felt their sleep was restless during the past week | | | |  |  |
| pscedd | Psych | Whether was happy much of the time during the past week /R | | | | |  |
| pscede | Psych | Whether felt lonely much of the time during the past week | | | |  |  |
| pscedf | Psych | Whether enjoyed life much of the time during the past week /R | | | | |  |
| pscedg | Psych | Whether felt sad much of the time duing the past week | | | |  |  |
| pscedh | Psych | Whether could not get going much of the time during the past week | | | | |  |
| hehelf | General | Self-reported general health | |  |  |  |  |
| heeye | General | Self-reported eyesight (while using lenses if appropriate) | | | |  |  |
| hehear | General | Self-reported hearing (while using hearing aid if appropriate) | | | | |  |
| hefla | General | Whether fallen down since last interniew | | |  |  |  |
| hefrac | General | Whether has fractured hip | |  |  |  |  |
| heji | General | Whether had joint replacement | |  |  |  |  |
| mmpain | General | Timed walk: whether had pain whilst walking | | |  |  |  |
| cfdatd | Memory | Whether correct day of month given | |  |  |  |  |
| cfdatm | Memory | Whether correct month given | |  |  |  |  |
| cfdaty | Memory | Whether correct year given | |  |  |  |  |
| cfday | Memory | Whether correct day given | |  |  |  |  |
| cfmem | Memory | Whether prompt given for prospective memory test (remembering  to write initials) | | | | | |
| cflisenq | Memory | Refers to cflisen |  |  |  |  |  |
| cfaniq | Memory | Refers to cfani |  |  |  |  |  |
| cflisdq | Memory | Refers to cflisd |  |  |  |  |  |
|  |  |  |  |  |  |  |  |

**Correlation matrices**

Table S10. Pairwise correlations of the Frailty Index across the 5 waves in LBC1936

|  | Frailty wave 1 | Frailty wave 2 | Frailty wave 3 | Frailty wave 4 | Frailty wave 5 |
| --- | --- | --- | --- | --- | --- |
| Frailty wave 1 | 1*** |  |  |  |  |
| Frailty wave 2 | .80*** | 1*** |  |  |  |
| Frailty wave 3 | .71*** | .81*** | 1*** |  |  |
| Frailty wave 4 | .65*** | .71*** | .79*** | 1*** |  |
| Frailty wave 5 | .57*** | .62*** | .74*** | .79*** | 1*** |

1. _*** all_ *_p_* _< 0.001_

Table S11. Pairwise correlations of the Frailty Index across the 5 waves in ELSA

|  | Frailty wave 1 | Frailty wave 2 | Frailty wave 3 | Frailty wave 4 | Frailty wave 5 |
| --- | --- | --- | --- | --- | --- |
| Frailty group 1 | 1*** |  |  |  |  |
| Frailty group 2 | .79*** | 1*** |  |  |  |
| Frailty group 3 | .71*** | .79*** | 1*** |  |  |
| Frailty group 4 | .61*** | .68*** | .75*** | 1*** |  |
| Frailty group 5 | .55*** | .59*** | .64*** | .73*** | 1*** |

*** all *p* < 0.001

**Sensitivity analysis**

We tested the effect of loss of data by including the same sample across all 5 waves in LBC1936 and we saw that effect does not change. This sensitivity analysis was the same analysis as before but ran only on Sensitivity analysis on 402 longitudinal participants.

Table S12. Results of multiple linear regression analyses showing associations between the optimal frailty PRS and the Frailty Index in the LBC1936 on 402 participants who took part at every wave – longitudinal data.

| **Multiple Linear Regression** | **β** | **SE** | ***p*** | **PRS R^2^** | **N** |  |
| --- | --- | --- | --- | --- | --- | --- |
| Frailty PRS at ~70 | .11 | 0.05 | **<.05** | .012 | 402 |  |
| Frailty PRS at ~73 | .12 | 0.05 | **<.05** | .013 | 402 |  |
| Frailty PRS at ~76 | .10 | 0.05 | 0.05 | .010 | 402 |  |
| Frailty PRS at ~79 | .10 | 0.05 | **<.05** | .015 | 402 |  |
| Frailty PRS at ~82 | .13 | 0.05 | **<.01** | .014 | 402 |  |

There was a significant difference in participants at baseline frailty levels between all participants (mean .16, SD = 0.09, N = 1005) and participants who dropped out after wave 1 (mean = .19, SD = 0.09, N = 205), *p* < 0.001. Multiple linear regression were then ran in the samples of participants who dropped out. The output for these is displayed in Table S13 and Figure S5.

Table S13. Results of multiple linear regression analyses showing associations between the optimal frailty PRS and the Frailty Index in LBC1936 participants who dropped out.

| **Multiple Linear Regression** | **Β** | **SE** | ***p*** | **PRS R^2^** | **N** |
| --- | --- | --- | --- | --- | --- |
| Frailty at Wave 1 (dropped out after Wave 1) | .14 | 0.07 | **<.05** | .021 | 205 |
| Frailty at Wave 2 (dropped out after Wave 2) | .18 | 0.08 | **<.05** | .035 | 140 |
| Frailty at Wave 3 (dropped out after Wave 3) | .08 | 0.09 | 0.3 | .005 | 138 |
| Frailty at Wave 4 (dropped out after Wave 4) | .22 | 0.09 | **<.05** | .043 | 129 |
|  |  |  |  |  |  |
