# Supplementary material for: Validation of a polygenic risk score for frailty in the Lothian Birth Cohort 1936 and English longitudinal study of ageing
Source: Sci Rep. 2024 Jun 1;14:12586. doi: 10.1038/s41598-024-63229-y (PMC11143351; doi:10.1038/s41598-024-63229-y)
Supplement: Supplementary file 3 — Supplementary Figure S2. [file 41598_2024_63229_MOESM3_ESM.pdf]

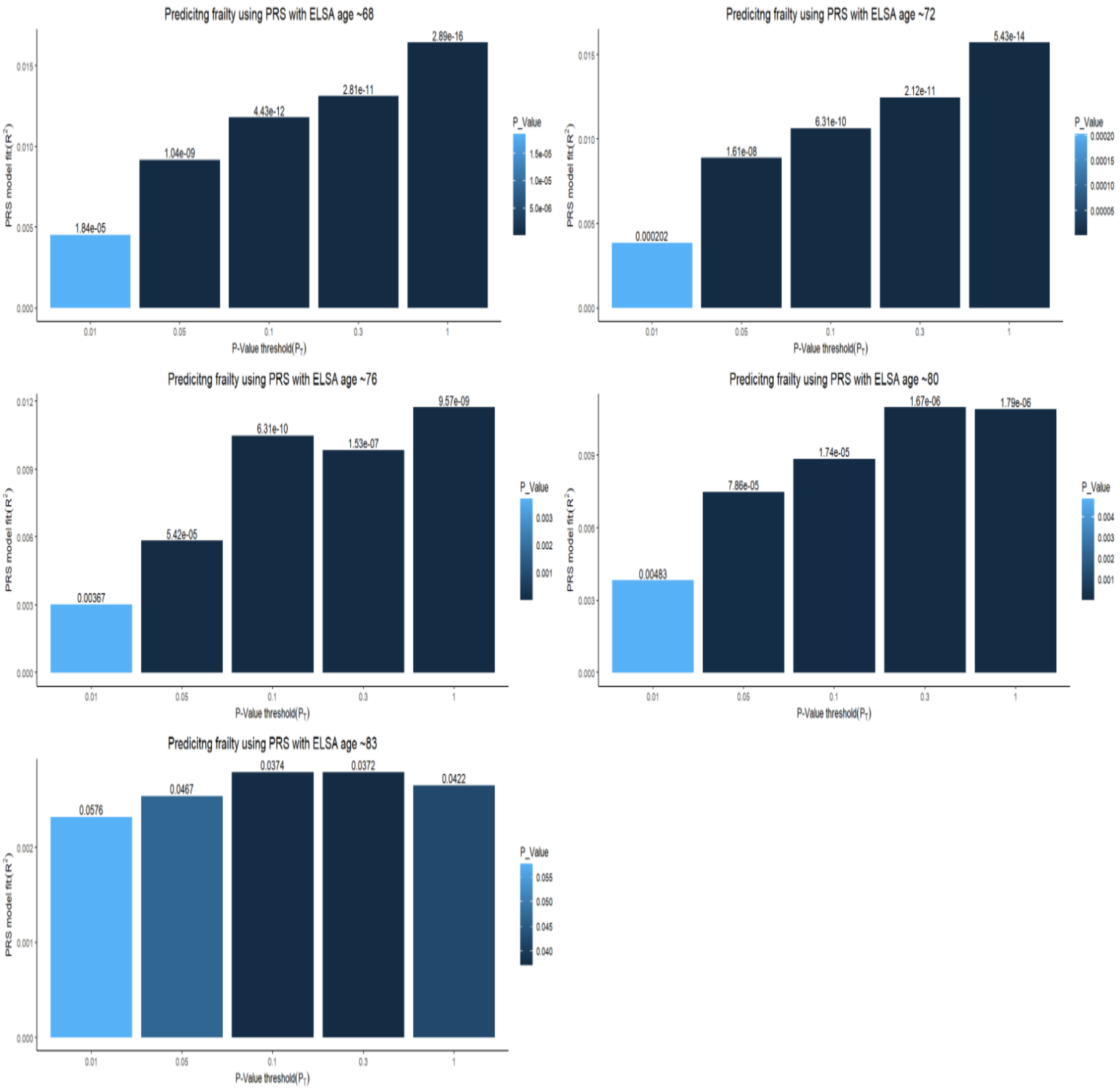

Figure S2. Multiple bar plots, from the multiple regression models, showing the optimal p-value thresholds when predicting frailty using PGS at five time points the ELSA. The x axis displays the varying different p-value threshold levels. The y axis displays the variance explained by the PGS. The values above the bar are the p-values from the regression output. The darker and taller the bar the stronger the prediction of the frailty PRS.
