# Supplementary material for: Validation of a polygenic risk score for frailty in the Lothian Birth Cohort 1936 and English longitudinal study of ageing
Source: Sci Rep. 2024 Jun 1;14:12586. doi: 10.1038/s41598-024-63229-y (PMC11143351; doi:10.1038/s41598-024-63229-y)
Supplement: Supplementary file 4 — Supplementary Figure S3. [file 41598_2024_63229_MOESM4_ESM.pdf]

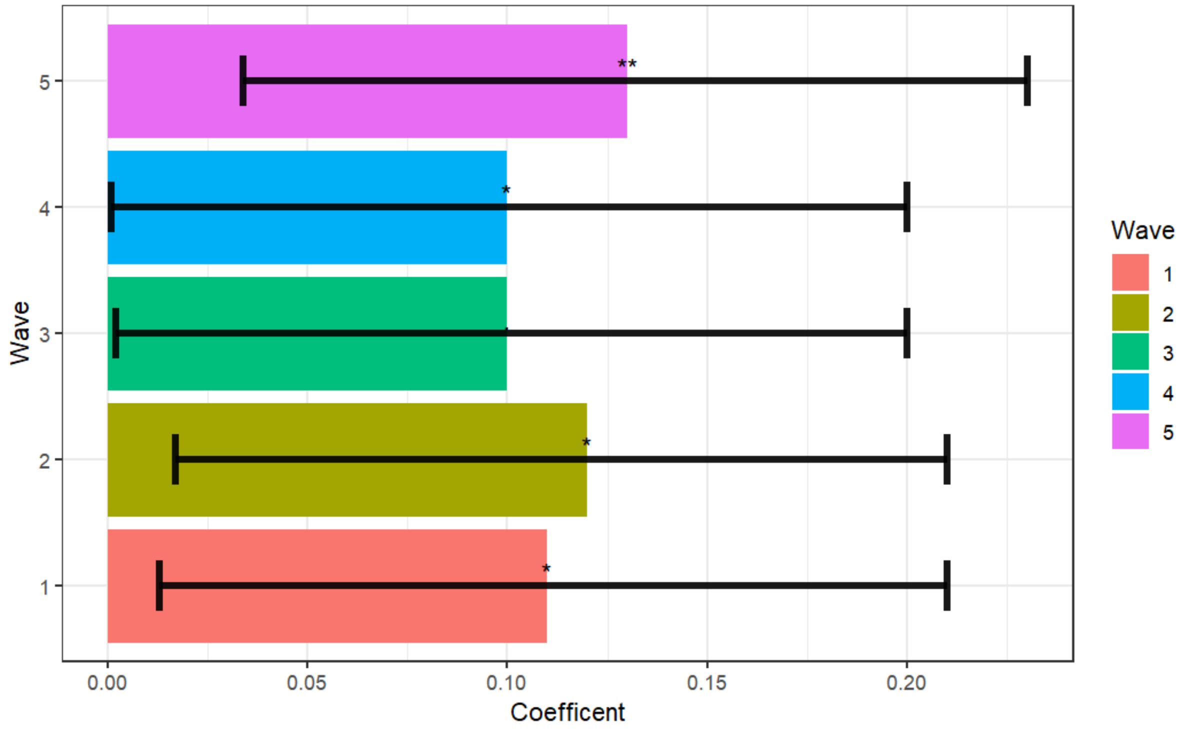

Figure S3. A bar plot comparing the standardized coefficients from the 5 waves on the same sample of all 402 participants in LBC1936.
