# Supplementary material for: Validation of a polygenic risk score for frailty in the Lothian Birth Cohort 1936 and English longitudinal study of ageing
Source: Sci Rep. 2024 Jun 1;14:12586. doi: 10.1038/s41598-024-63229-y (PMC11143351; doi:10.1038/s41598-024-63229-y)
Supplement: Supplementary file 5 — Supplementary Figure S4. [file 41598_2024_63229_MOESM5_ESM.pdf]

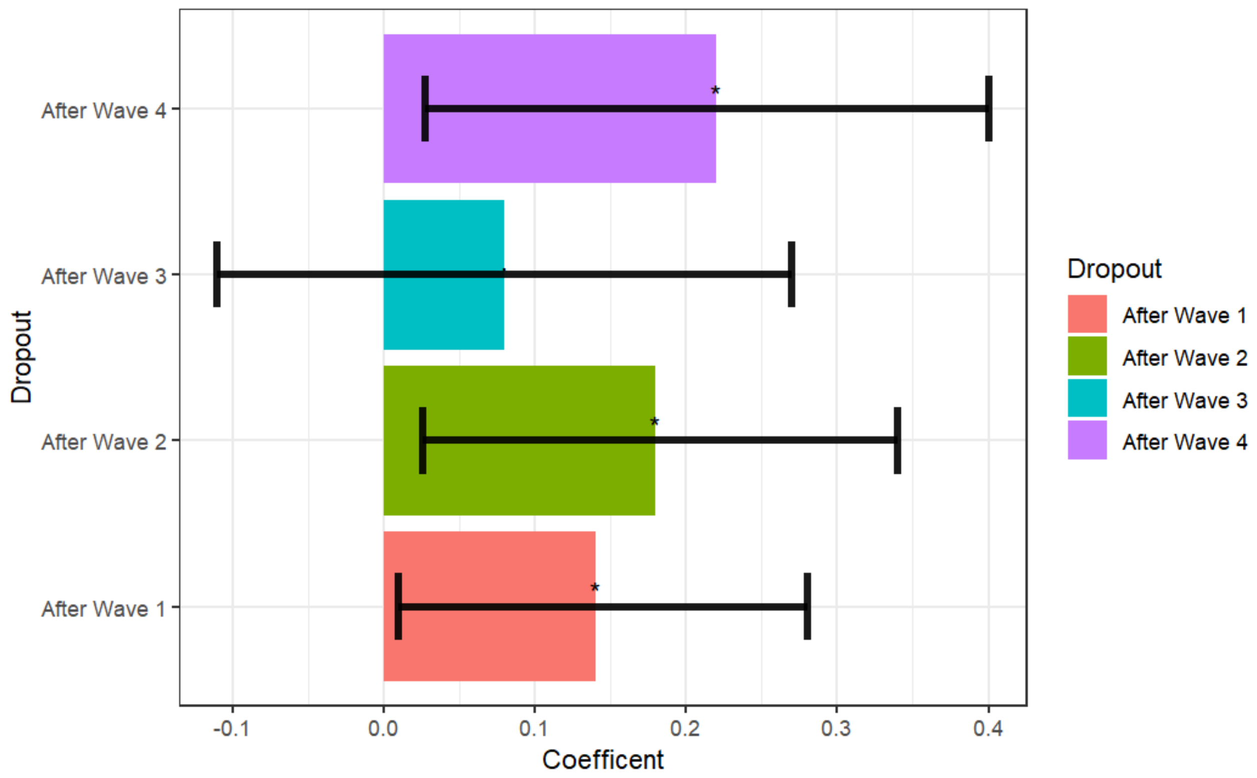

Figure S4. A bar plot comparing the standardized coefficients from participants who dropped out.
